# Embryology of the Conduction System for the Electrophysiologist 

**Published:** 2010-08-15

**Authors:** Sultan Mirzoyev, Christopher J McLeod, Samuel J Asirvatham

**Affiliations:** 1 Mayo Medical School; 2 Department of Internal Medicine, Mayo Clinic, Rochester, Minnesota; 3 Division of Cardiovascular Diseases, Mayo Clinic, Rochester, Minnesota; 4 Department of Pediatrics and Adolescent Medicine, Mayo Clinic, Rochester, Minnesota

**Keywords:** embryology, cardiac conduction system, arrhythmia, electrophysiology

## Abstract

It is critical for interventional electrophysiologists to thoroughly appreciate the topographic and developmental anatomy of the heart and its conduction system. Not only is understanding cardiac anatomy important to prevent complications from collateral damage and to help guide catheter placement, but developmental anatomy allows a deeper appreciation of the arrhythmogenic substrate. In this article, we briefly review the relevant stages of cardiac development for electrophysiologists. The potential location of normal and abnormal conduction patterns resulting from heterogeneous developmental origin is discussed.

## Introduction

The cardiac conduction system is a complex and highly specialized network that is fundamental to cardiac electrophysiology. An understanding of the embryology underpinning the development of the mature heart not only offers insight into the critical spatial relationships of the conduction system but enables conceptualization of the relevant structures and their variants. In addition, the structure of the walls and fiber orientation is such that conduction of electrical activity is favored along developmental planes and tracts without the need for discreet conduction tissue. Furthermore, and critical for the ablationist, structures physiologically unrelated to each other come to lie in close proximity - in a manner that is not immediately intuitive. In addition, the origins of atrioventricular (AV) reentry, AV node reentry, atrial fibrillation and ventricular tachycardia often have a clear anatomical basis. Hence, in order to fully grasp these functional and anatomical considerations, one needs to begin at a cellular level and follow the formation of normal cardiac anatomy from the tubular primordial heart.

## Myocyte development and fiber orientation

During cardiogenesis, myocytes develop into either contractile or conduction cells. Three models have been proposed by which cardiac cells develop and differentiate [1]. The first model has been traditionally adopted by electrophysiologists and is based on a multiple ring theory. It hypothesizes that during heart chamber development and growth, cells in certain regions of the heart tube do not proliferate as rapidly as cells in genetically predetermined atrial and ventricular regions. As the tubular heart grows, the slower-proliferating myocytes form constrictions or rings around which the heart will fold.

A second recruitment model is based on the idea that the conduction system framework is present in early development and enables recruitment of adjacent myocytes to form further elements of the conduction system.

The third model, the early specification model, postulates that myocytes begin expressing either conduction genes or working (contractile) genes early in the development. Cells expressing conduction system markers slowly proliferate and form components of the conduction system, whereas cells lacking the markers proliferate faster and develop into contractile tissue.

**Figure 1:**
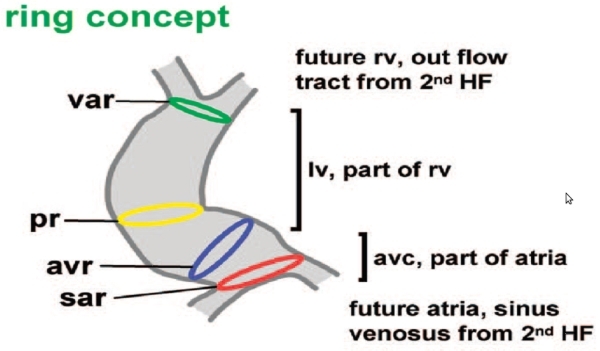
The embryology of the cardiac conduction system mirrors the development of the heart.  A common view used by electrophysiologists is the ring concept.  Here differential rates of proliferation result in constricting band-like segments often representing the future sites of the conduction system. Figure courtesy of Antoon F.M. Moorman, Academic Medical Center, Amsterdam

**Figure 2:**
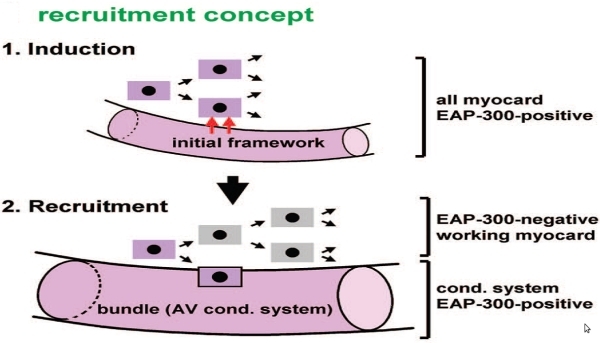
The recruitment concept is another framework used to explain cardiac and conduction system development.  Here induction of conduction versus contractile cells results in recruitment of the appropriate cell type to the future anatomic location in the mature heart. Figure courtesy of Antoon F.M. Moorman, Academic Medical Center, Amsterdam

**Figure 3:**
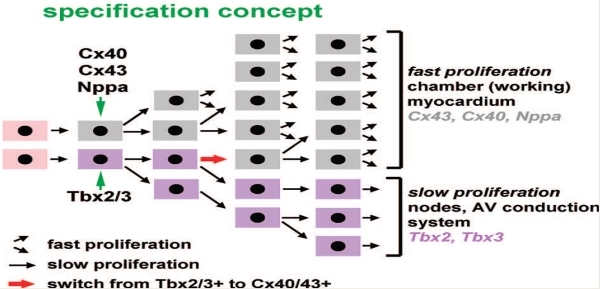
The specification concept is in some ways a hybrid of the ring and recruitment concept.  See text for details. Figure courtesy of Antoon F.M. Moorman, Academic Medical Center, Amsterdam

Putatively, the development of the conduction system can also continue throughout life, perhaps explaining why one can find fascicle-like tissue in some unusual locations (Figure 1,2 and Figure 3). The electrical action potential originating in these regions spreads between cardiac cells through gap junctions that are concentrated at the ends of the cardiac myocyte [2]. The rapid electrical communication between cells is facilitated by the proteins (connexins) within the gap junctions that act as micro-channels and allow the passage of ions between cells. This electrochemical and metabolic coupling produces depolarization of cardiac muscle and facilitates intramyocardial conduction. Although this arrangement allows the heart to contract as a unified syncytium, preferential conduction occurs in the longitudinal axis as gap junctions are concentrated at the ends of the cardiac myocyte [3,4].

**Figure 4:**
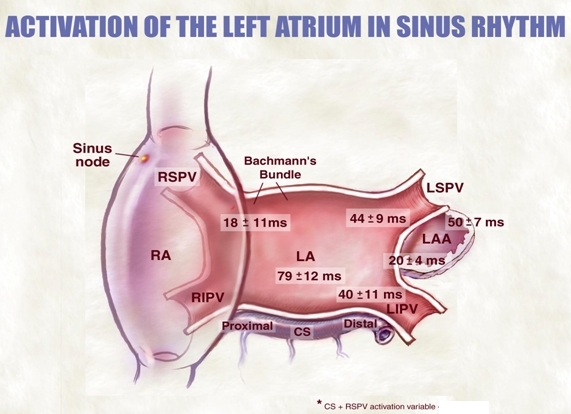
In addition to understanding the development of the conduction system per se, the construction of the fiber orientation of the contractile myocardium is relevant for electrophysiology.  For example, as a result of preferential fiber orientations (Bachman bundle, peripulmonary vein, etc.), conduction occurs in a specific ordered pattern.  The principal electrical conduction between the right and left atrium is Bachman's bundle, and from here relative early excitation of the periappendage region and the pericoronary sinus region occurs with the late activation in the posterior left atrium.

Four major connexins have been identified in the human heart, and based on their conductive properties they are differentially expressed and located throughout the heart. Fast-conducting cardiac tissues and atrial muscle predominantly express Cx43, while Cx45 is seen chiefly in the slow-conducting pathways, including the sinoatrial node and AV node [5-7]. Fiber orientation of the cardiac structures is also critical for the understanding of electric impulse propagation delays. For example, the left upper pulmonary vein contains circumferential fibers at the ostium, whereas some portions of the left lower vein contain longitudinal fibers. The circular fibers will create more delay and will be easier to observe and see pulmonary vein potentials.  Because longitudinal fibers of the Bachmann's bundle go from the right atrial appendage to the left atrial appendage, left atrial appendage tachycardias may appear earlier in the right atrium. However, posterior wall of the left atrium is activated even later than the regions near the left atrial appendage (Figure 4).  The orientation of muscle fibers also plays a role in appearance of double potentials on the crista terminalis, which has vertical fibers while all the pectinates are horizontal.

## Chamber and Large Vessel Development

### The atria

The tubular primordial heart is formed during early cardiogenesis from the fusion of two endothelial strands of cardiac mesoderm [8]. Each strand is continuous cranially with a dorsal aorta (the outflow tract), and caudally with a vitteloumbilical vein (the inflow tract). This cylindrical structure subsequently loops and segmentally dilates, hence forming the segments, the basis of the cardiac chambers. After looping, several segments exist and are connected at 'transitional zones' [9]. The sinus venosus, which forms the venous inflow chamber, the ventricular inlet segment, and the outlet segments ultimately septate and divide to form the AV valves and outflow tracts of the mature heart. This combination of tubular growth, looping, and expansion of the cavitary myocardium, is the basic initiating step [1] and like all steps in the developing organism is driven by a cascade of regulatory genes and transcription factors. It is important to note that concurrent with the basic chamber development, separate transcription factors simultaneously coordinate the development of the cardiac conduction system [10]. The first indication of cardiac contraction occurs around 23 days after conception in the human and peristalsis of the tubular heart [11]. Primitive coordinated and sequential chamber contraction occurs soon after looping of the heart and is subsequently initiated by the primordial atrium which acts as the interim pacemaker of the heart. Following that, the sinus venosus adopts this function, finally to be usurped by the sinoatrial node develops during the fifth week [12].

During the first and second month of embryonic development, continued looping of the heart moves the ventricles inferior to the atria, and atrial and ventricular septa begin to form. The atrial septum forms in several stages and partitions the atrial chambers. Initially, a fold of endocardium called septum primum forms, leaving an opening called the ostium primum between two atria. However, as septum primum continues to grow, the ostium primum decreases in size and eventually closes, yet not until a second aperture called the ostium secundum has begun to develop. Both openings function to allow shunting of blood from right atrium to left and bypass the fetal lungs. In concert with this, another septal wall begins to form: the septum secundum. This thicker and more muscular wall grows adjacent to the septum primum and ultimately fuses to form the complete atrial septum with its muscular character derived from the septum secundum. The foramen ovale is left as a remaining hiatus and is continuous with the ostium secundum - allowing shunting of blood from right atrium to left atrium  [13]. It is important to recognize that only the central portion of the septum that is truly part of the inter-atrial septum. The posterior septum is really the pyramidal space and is outside the heart. This is important for electrophysiologists and is the reason why the P wave is wide whenever there are arrhythmias coming from the posterior pyramidal space like a posteroseptal tachycardia or atypical AV node reentry.

### Left atrium and pulmonary veins

As the primitive heart tube grows and partitions, certain regions of atrial and ventricular tissue stem from different origins. Right and left smooth arterial posterior wall regions arise from venous structures, and most of the sinus venosus is incorporated into the right atrial smooth posterior region. The pulmonary veins develop as four separate blood vessels in the mesenchyme of the mediastinum and are incorporated in the left atrium  [14]. The pulmonary veins give rise to the posterior region of the left atrium, yet right and left atrial appendages are derived from the primordial atria  [15]. The arrhythmogenic properties of pulmonary veins appear to be related to their 'myocardialization', an ill-defined process involving the growth of myocytes into the mesenchymal projections or migration of myocardial cells into this region  [16,17].

Although the underlying mechanism is not clear - the pulmonary veins develop a myocardial sleeve, which are longest in the left superior pulmonary vein and shortest in the right inferior pulmonary vein  [18]. Interestingly, it also appears that the arrhythmogenicity noted clinically for each pulmonary vein corresponds to the length of the circumferential myocardial sleeve  [19]. Furthermore, the type of myocyte within the pulmonary vein may themselves be more liable to develop arrhythmia, evidenced by the presence of pacemaker P cells and Purkinje cells seen predominantly in the pulmonary veins of those patients with atrial fibrillation  [20]. The characterization of pulmonary vein myocytes has also identified discrete electrophysiological properties, distinct from atrial myocardium. Different resting membrane potentials, action potential amplitudes, phase 0 upstroke velocities, and  action potential duration all support the notion that these myocardial sleeves are different functionally  [21], yet whether these properties are fundamental to the arrhythmogenicity of these structures remains to be confirmed.

### The great vessels

As the embryonic tube gradually transforms into a four chamber heart, the most cephalic end of the outflow - the aortic sac - divides into the aorta and pulmonary trunk. Extracardiac neural crest cells migrate to the heart and contribute to aorticopulmonary septation  [22]. The semi-lunar aortic and pulmonic valves develop as localized swellings of endocardial cushions after mesenchyme surrounding the vessels fuses with endocardial tissue  [23].

### The ventricles

During development, the left ventricular wall is composed of two layers: the outer condensed myocardial layer and the inner layer containing multiple trabeculae. It is within the inner layer, a non-dense muscular ridge composed of trabeculae with intertrabecular spaces is located. Although there is still scarce information available on the normal development of papillary muscles, current evidence supports the concept that these erupt as a small myocardial ridge in the left ventricle which will then develop into papillary muscle of the mitral valve  [24].

Left ventricular false tendons are thin fibrous or fibromuscular bands that extend across the left ventricle between the interventricular septum or papillary muscle and the ventricular wall  [25,26]. These abnormal cardiac structures are believed to rise from the inner muscular layer of the primitive heart  [25,27]. Furthermore; false tendons can play a role in cardiac arrhythmias. In fetal, newborn, and infant hearts, false tendons are primarily composed of heart muscle tissue and some connective tissue in different proportions. Some false tendons contain cardiac conduction tissue and are infrequently connected to the left branch of the AV bundle, therefore contributing to reentrant ventricular arrhythmias  [28-30].

## Specialized conduction tissue development

It was thought for many years that the specialized conduction tissues of the heart were of extracardiac neural crest origin and that migration into the myocardium was integral  [31]. Although neural crest cells do migrate into the heart, particularly around the great arteries and AV septum and may be of electrophysiological consequence from their autonomic ganglia inputs, the conduction system has been shown to develop through the differentiation of cardiomyocytes  [32]. The AV node itself, which lies on the right side of the interatrial septum, at the apex of the Triangle of Koch, is a likely downstream consequence of precursor cells in myocardium within the embryonic AV canal  [33-37]. Moreover, based on the identified phenotypic similarities between AV nodal cells and primitive cardiomyocytes  [38,39], it has been hypothesized that slow pathway region surrounding this structure is a result of primary myocardial cells being inhibited from maturation  [38,40,41]. The penetrating bundle of His has no myocardium in its immediate vicinity and is instead insulated through the maturation of fibroblasts and fibrous tissue. It may also be that the migration of neural crest cells into the heart facilitate the development of the insulation as some of these cells possess similar markers to glial cells  [42].

Distal to the His bundle, the Purkinje fibers also develop through the differentiation of cardiomyocytes yet are intimately linked to the influence of epicardium-derived cells (EPDCs). These cells migrate into the heart from the epicardial surface of the embryo and are integral to the development of the connective tissue that forms the fibrous heart skeleton  [33,34]. Although not entirely clear at present, it appears they may produce inducing factors which encourage Purkinje fiber growth  [45].

## Accessory pathways

In a normal, mature heart, upper chambers are physically separated from the lower chambers by a region of fibrous tissue, leaving the bundle of His as the solitary connection between atria and ventricles  [46]. Integral to the development of this insulation is the annulus fibrosis which is formed from the fusion of sulcus tissue during early atrial and ventricular folding  [47]. Accessory pathways occur on the AV annulus and are always exterior or epicardial  [47] to the fibrous annulus. The presence of these aberrant myocardial bundles confers ventricular pre-excitation through early activation of the ventricle and provides a potential circuit for the development of the AV reentrant tachycardias  [48]. Most of the accessory pathways are fast conducting pathways as in the case of the bundle of Kent in Wolff-Parkinson-White syndrome; therefore, they are believed to be derived from chamber myocardium. However, in rare cases the arrhythmia is caused by slow conducting fibers of AV-nodal character as in the case of Mahaim tachycardia. These 'slower' conducting structures are not duplicate AV nodes, but probably remnants of AV node-like tissue found all along the AV annulus that insert into the right ventricular free wall or right bundle branch around the lateral aspect of the tricuspid valve  [47,49]. Interestingly, gene expression studies correlate these with the development of the right ventricular inflow and moderator band, the morphological correlates of Mahaim or atriofascicular pathways  [50].

Rarely, accessory pathways linked to the true AV conduction system, such as fasciculoventricular or nodofascicular tracts, may be due to failure of transcription factors during His bundle and right bundle development  [51]. The fibrous insulation of the conduction tract is not completed in its entirety and there is early excitation of the ventricle through the conduction system. Thus, in these patients there is early activation of the base of the ventricle, even though there is no true AV connection.

## Coronary sinus

Although the coronary sinus (CS) is not a true part of the cardiac conduction system, an understanding of this structure is vital for the electrophysiologist. The right and left horns of the sinus venosus develop into the superior vena cava and CS respectively. The latter develops as the left superior vena cava degenerates and hence  is dilated in maldevelopment and maintenance of this early structure  [52]. Diverticula may rarely also develop in this process and potentially can affect the electrical insulation of the AV groove, in which the CS lies.  Hence, the majority of the cases occur in combination with the presence of aberrant AV conduction pathways - believed to perhaps account for the supraventricular tachycardia or even sudden cardiac death that is seen in some of the CS diverticulum cases  [53]. It is likely that during cardiogenesis, defects in the wall of CS contribute to the development of the diverticuli, which are composed of myocardial tissue, thus serving to bridge the AV boundary as accessory pathways  [54,55].

## Conclusion

As the developmental origins of the cardiac conduction system continue to be deciphered, further insights into the mechanisms of arrhythmia will be gained. For the electrophysiologist this pathogenesis is invaluable, as is the anatomy, which can only be truly understood by tracing the relationships from their origins.
